# Professional Ollapodrida

**Published:** 1859-08

**Authors:** 


A T L A N T A

Medical and Surgical Journal.

VOL. IV.] AUGUST, 1859. [No. XII.

### ORIGINAL COMMUNICATIONS.

### ARTICLE I.

*Professional Ollapodrida.*—BY A RETIRED PHARMACIEN.

*Syrup of Galls* is a nice and effective prepartion for internal use in the diarrhœa of infants, and may be added to chalk mixtures to increase their astringency in prefernce to the astringent tinctures. To prepare the syrup:

℞.—Coarsely powdered Galls, a half ounce
Powdered Cinnamon and Mace, *aa.* two drams.
Brandy, a halt pint.
White Sugar (in lumps), three ounces.

Place the powdered drugs and brandy in a cup—arrange a grating of clean iron wire across the top, upon which the lumps of sugar are to be placed and the brandy set on fire. When cold carefully pour off the syrup clear, and bottle for use. Dose for an infant 10 to 20 drops.

*Tannic acid*, a very good astringent pill for adults is:

℞.—Tannic acid

Sulphate of zinc, *aa*, 32 grains.

Powdered Opium, 16 grains

Syrup of gum arabic, sufficient.

Make into 32 pills and direct one after every discharge from the bowels in diarrhœa.

The remarkable solvent power of glycerine for Tannic acid may be resorted to with advantage when it is desired to secure a powerful astringent or styptic effect.

℞.—Tannic acid.

Glycerine, *aa*, 1 ounce. Dissolve.

A bit of sponge wet with the solution and passed up to an ulcerated and bleeding os uteri, restrains the hemorrhage satisfactorily. The various applications of a compound so powerfully astringent will readily suggest themselves; diluted with an equal bulk of water, it is used for hardening the nipples.

*Pile Ointment.*—℞.—Tannic acid, 2 drachms.
Sulphate Morphia, 6 grains.
Glycerine, 2 drachms.
Lard, 1 ounce.

Rub the acid----Morphia and Glycerine together in a mortar, add the lard and mix thoroughly.

*Cotton Root* when fully matured, contains a variety of tannic acid differing from the acid of galls, as does the tannin of kino, catechu, rhattany, &c. A tincture may be prepared from it and used as a substitue for these several astringents—observing that the Cotton Root is said to produce abortion and should not be used when such a result is possible.

*Goulard's Cerate* is not a preparation proper to be *kept* upon the shelves of a physician. Under no circumstances should it be used as a *soothing* application to a blistered surface, unless freshly prepared for the purpose. A case of lead colic in a child was clearly attributable to the ill-advised application of this cerate to the blistered abdomen.

*Peruvian Bark.*—Of all the simple pharmaceutical preparations of bark, the *Fluid Extract* is perhaps the most elegant and generally eligible. It represents the full remedial value of the drug in a concentrated and more palpable form—while it exhibits the alkaloids in their state of native combination. It has proved itself peculiarly serviceable in obstinate nurse's sore mouth. The solid extract of Cinchona-Rubra is a convenient excipient and adjuvant in combination with iron by hydrogen—although the cincho-tannic acid present in the bark is incompatible with the iron powder—yet the amount of moisture usually contained in the extract is probably insufficient to oxidize the iron so as to permit the combination with it—in practice the compound is found to be unobjectionable. The precipitated extract is entirely free from the incompatibility.

*Lupulin.*—The fluid extract of Prof. Proctor is the most eligible form; it should be given upon a lump of sugar—for when taken in water, in consequence of its oleoresinous character, it floats upon the surface and adheres to the mouth and tongue, leaving a long persistent taste of the drug behind.

*Wild Cherry.*—The syrup prepared in accordance with the last officinal formula, should be upon the shelf of every physician who has not an expert apothecary at his hourly command; as a general basis for an elegant cough mixture it is unrivaled; add to it as your case suggests, whether antimonial, ipecac, nitrate potass, morphia or other opiate or anodyne, and give a teaspoonful or tablespoonful as you may desire the effect of the hydrocyanic acid. The patient secures in a somewhat similar preparation--- all the efficacy of Ayer's Cherry Pectoral without its objectionable features. In preparing the syrup, use the bark of the root only, and do not rely on the druggist for your supply, but have it procured from the woods or fence corners in the fall, dried in the shade----ground to a fine meal, made into syrup, bottled and sealed for the twelve months supply. The syrup keeps more perfectly when tightly bottled, though the bark will do even in a close drawer. Allow the bottles to stand for a month and pour off into clean ones the bright and transparent syrup, carefully excluding the druggy portion, condense the drugs into as few bottles as possible and after a week pour off as before, until but little is left to be discarded. In making this syrup, unless the officinal directions for maceration, percolation and solution of sugar without heat are strictly followed the product is worthless, and further, no iron untensil unprotected by tin should be used in the process.

*Aromatics.*---If an orange or lemon be rubbed upon a section of loaf sugar and the stained sugar scraped off and closly bottled, a nice flavoring material is obtained which may be added with advantage to sundry powers and mixtures to disguise a nauseous taste; such a material is decidedly more palatable and fruity than any preparation from the volatile oil.

*Essential Oils*, by keeping, absorb oxygen from the air and deposit a class of camphory bodies termed stearoptens, while the delicacy of odor and flavor is lost and a rankness developed, these changes are greatly hastened by exposure to a strong light. To keep these oils in good condition, procure the stock as fresh as possible, add an equal bulk of high proof cologne, spirit or inodorous alcohol and keep the mixture in glass stoppered bottles in a dark and cool place, or if upon the shelf, surround the bottle with thick brown paper. Much of the essential oils now sold are already "fixed up" with the alcohol, not so much it is to be feared for the sake of the keeping quality, as to enhance the profit upon the drug. This adulteration may be much better tolerated than the former vile practice of adding spirits of turpentine.

*Ague Pill.*----A very cheap and at the same time efficient preparation is:

℞.---Sulphate Cinchonia, 3 drachms.
Powdered extract Liquorice, 30 grains.
Oil Black Pepper, 1 drachm.

Mix, make 64 pills, and use 3, 4 or 6 as may be required to arrest the paroxysm. The cost of material for the 64 pills need not exceed 20 or at most 25 cents.

*Ginger.*—A country merchant recently bought 10 pounds of E. I. Ginger, out of which he culled roots to the amount of 2 or 3 pounds, evidently not ginger. Upon examination the adulteration proved to be Tubers of Madras and Bengal Turmerics mixed with those of the round Zedoary.

A "highly concentrated essence or fluid extract of the best Jamaica ginger, prepared upon the most scientific and refined principles of chemistry and pharmacy---carefully retaining all the valuable and healing qualities of the root," &c., &c., has become a household institution in our land--- and really fills a useful place upon the physicians shelves. It may be purged of all odor of quackery and prepared *secundem artem* by the following method.

Take of Jamaica ginger 3 pounds, grind to a meal and pack properly in a funnel or displacer, covering the surface with cloth or paper, pour on high proof alcohol and percolate one gallon for use, after which another gallon may be displaced to fully exhaust the root, and set aside to be used as menstruum, at the next repetition of the process. If it be desired to make an essence of a rich, deep, brandy color to attract the eye, the hard, brittle and highly resinous kinds of the unbleached root will be selected; on the other hand, if the intrinsic worth be the object, the softer, more oily and pungent kinds will have preference; if, again, money saving be the sole object, the cheapest kind will be selected by the quack and used with a sparing hand, supplying color with caromel (burnt sugar) and pungency with cayenne. Essence of Ginger is not only a valuable internal stimulant, but is also an active rubefacient when applied upon a flannel to the surface---it is a "Pain Killer" of the better sort, better internally, better externally, and the bottle even is quite as potent as that from the hands of "Perry Davis" himself.

*Nitrate of Silver.*--A convenient and generally unobjectionable excipient, for this salt in pill, is the firm white turpentine found concreted upon the pines in our own forests; the salt is powdered and incorporated with a smaller or larger quantity of the turpentine, as may be desired, and rolled into pills with milk sugar or starch powder for the coating. If the turpentine be carefully selected and the hands entirely pure, the pills will be white and handsome.

Every body does not know that a stick of caustic may have its end formed into a globular, blunt pointed or quite sharp pointed extremity at pleasure by simply turning it upon a heated silver coin held in a pair of forceps. The practice of attempting to sharpen a caustic pencil with a penknife, scattering the dust upon the table and hands, is not only unsatisfactory but filthy.

*Sub-nitrate of Bismuth.*—Last fall Prof. Rogers, in a report to the College of Physicians of Philadelphia, stated that in the progress of a case of arsenical poisoning, it became necessary to determine the purity of sub-nitrate of bismuth. He examined ten samples, from different sources, some of which were imported, and all were found to contain arsenic, with the exception of two—the amount he did not determine, but infers it to be small—and doubtless due to a want of care in washing the precipitate.

*Sub-carbonate of Bismuth*, placed before the profession some two years ago, by Dr. Hannon, of Brussels, has not realized fully the expectations raised in the mind of thi present writer, although he still uses it in preference to the sub-nitrate—yet not in the large doses (15 to 45 grains and upwards,) recommended by Hannon. It is easily prepared from the sub-nitrate, by dissolving in nitric acid, and precipitating with a solution of carbonate of soda; well washing the precipitate to free it of all vestages of arseniac acid.

*Pyrophoric Iron.*—Take an ordinary test tube, fitted with a good cork, pass down tightly through the cork a small glass tube, and draw off the end of the latter to a capillary opening by softening it in a spirit lamp frame. Now fill the test tube one-third full of oxalate of iron, adjust the cork, and slowly heat the oxalate to expel first the water of crystalization, and afterwards to decompose it, shaking the tube occasionally to expose fresh portions to the heated surface, until the whole is uniformly black throughout, then fuse the end of the capillary tube with the lamp or a jet from the blow-pipe, and lay aside until perfectly cold. If the heat has not been too great, or long-continued, when the cork is removed, and the iron powder poured out, it will instantly take fire, to the astonishment of the uninitiated.—This experiment is a pleasing one to an enterprising student in the office of a country practitioner, and generally succeeds in opening the eyes and mouths of the wonder-mongers lounging about "the shop."

*Oxalate of Iron* is easily prepared by precipitating a solution of sulphate of iron by means of a solution of oxalic acid ; both solutions should be saturated to avoid too much loss of the oxalate, which is somewhat soluble. An additional quantity may be thrown down by saturating the free sulphuric acid with soda, but it does not pay.

*Iron by Hydrogen* is an article which can with a little care be made more perfectly upon a small scale, than in the larger way as manufactured for market. An ingenious physician with a tube of hard German glass, two feet long, one inch bore, and a few pieces of smaller tubing of softer lead glass, together with a spirit lamp and wide-mouth bottle, may arrange an apparatus which shall supply him with two or four ounces of the iron powder of the very best quality, with a few hours attention. The sulphate of iron, oxalic acid, zinc scraps and sulphuric acid are of trifling cost. The advantage gained in the glass tube of being able to inspect the interior from beginning to end of the process, ensures with a little experience, always a perfect product, which is by no means the case in a large iron tube. In using the oxalate of iron, less hydrogen is required than with the subcarbonate, and the process is considerably hastened, while the product is much more impalpable, indeed, pyrophoric, unless the proper care shall be used to raise the heat at the close of the process sufficiently to prevent this.

*The Saccharated Carbonate of Iron* is an improvement upon the officinal Vallet's mass, in so far as its form, that of powder, fits it for more convenient portability, and it may be readily administered in pill, powder or electuary, while the mass is almost always so soft as to be only suitable for a linctus, requiring too mnch inert powder to form it into a pill.

*Hydrated-Sesqui-Oxide of Iron* is the antidote now most esteemed in arsenical poisoning. It is the *duty* of every apothecary—a duty which he owes to the public, and especially to his customers and medical patrons—to keep always on hand the materials ready to furnish the preparation at a moment's notice, and this freshly prepared, in which state alone is it to be at all relied upon. It was the practice of the writer in by-gone days to keep in a spot upon the shelf specially allotted for this purpose, accessible and familiarly known to all in the house, the proper measured quantities of solution of tersulphate of iron, of aqua ammonia and water, each in its bottle, labelled with the directions as to the use to be made of it in the process. With these stood a pitcher and deep dish, with straining cloth, and a wide-mouth bottle, corked and labelled with the directions for administering the antidote. The preparation could be made and delivered to the messenger in less than ten minutes. For an interesting and instructive paper upon this subject see the "American Journal of Pharmacy," for 1853, p. 104.

*Liquor Ferri Iodidi* may be prepared by every apothecary who is capable of compounding a prescription. One of the best forms for the iron used is very fine wire nicely cleaned with sand paper, coiled around a small glass tube or cedar pencil, and cut into small separate rings by a pair of scissors, one blade of which is passed into the coil, and the several turns of the wire cut through. The very fine wire possesses the advantage of presenting an extended surface for the action of the iodine, and ensures a high degree of purity in the material itself; since it is only the purest and best iron which can be drawn into these fine threads. The finest standard loaf sugar should be powdered and used, to avoid any necessity of exposing the syrup, either in clarifying or filtering it. It is both convenient and desirable (for physicians) to avoid decomposition from exposure; to put it up fresh in ounce vials, tightly corked, and having a clean iron wire passing down through the cork and nearly to the bottom of each vial. If a patient require four ounces, it is better in four vials than in one. Light does not injure it if air be excluded.

This remedy is incompatible with most of the vegetable tonics—it may, however, be eligibly combined with compound tincture of gentian, and if a more elegant and potent tonic adjuvant be desired, try the combination with sulphate of cinchonia as suggested in the May number of this journal, at page 533.

*Fluid Extract of Valerian* represents the entire therapeutical virtues of the root in the most desirable form for general use. It is not expensive, and every practitioner who employs the drug at all should have it in this form.

*Atwood's Deodorized Alcohol* of high proof, is the best; and costs in market but a few cents in the gallon above the ordinary article of equal strength; the difference in quality more than compensates a physician for the trifling additional cost.

The administration to the sick of *wines* (?), the purity of which is vouched for only upon the recommendation of the keeper of a local "doggery," or even the equally dubious assertion of a New York jobber, cannot be too carefully avoided. The writer relies upon good country whisky or peach brandy, in preference to five and eight dollar brandies from the north. There is now no limit to the cupidity and impudence of many, (perhaps the majority,) in the wholesale drugged liquor trade—no amount of money commands with *certainty* a pure liquor from their cellars. It is to be hoped, that the day is not far distant, when the hill-sides and mountains of Upper Geo., will supply us with an abundance of pure catawba wines and brandy from the hands of men of a more primitive honesty and integrity—whose souls are not buried head and ears in the folds of a filthy shin-plaster.

*Camphor.*—If a file or watch-spring saw be wetted with a solution of camphor in spirits of turpentine, it will cut through glass as smoothly and rapidly as through iron.—This is an *old* fact not generally known, which might benefit some body.

*Corks.*—Physicians should always select the very best velvet corks, regardless of any reasonable cost, and they should be of the taper form. The waste of time, and wear and tear of patience in fitting inferior ones pays very dearly for the supposed saving. In most apothecary establishments also, the loss of time of clerks, with the losses by imperfect stoppering, will usually counterbalance the cost of the best corks.

Corks may be fitted accurately to vials, tubes and flasks, by the skilful use of a very sharp knife, with a sawing rather than cutting movement; but the most convenient and sure mode is, by the use of a fine wood rasp, with as many flat and rat-tail files, of assorted sizes and qualities, as convenience and economy may dictate. A very cheap, and at the same time, efficient outfit for this purpose, is a half dozen sheets of assorted sand paper, upon which the corks are to be rubbed down to the required form, size and smoothness.

*Opium.*—It is said that an *old hard* opium pill will be retained and quiet an irritable stomach, when no other form of either opiate or anodyne of any kind can be borne. The effect upon the gastric nerves is so very gradual and gentle, on account of its slow solubility, as to be wholly unirritating and gradually lull the irritability of the organ, until rest and sleep result. A half grain each of opium and ipecac finely powdered and formed into a pill is, in most cases, a satisfactory substitute for dovers powder; and is much more conveniently carried in the pocket, while the dose is accurately weighed, and requires no guess-work. Two of the pills are equivalent to ten grains of the powder. In the use of dovers powder, we desire the *simultaneous* action of the two essential ingredients upon the economy. To secure this it is necessary that the constituents be intimately mixed, and also, that they be in a state of extreme comminution; hence the longer the trituration be continued the more perfectly satisfactory will be the preparation.

*Syrup of Codeia*—(codeine).—Formula long used by the writer—in preparing this syrup, is: ℞.—Codeia 24 grains, water 4 ounces; rub in a mortar until dissolved; add of white sugar 8 ounces, avoirdupois, and gently heat until dissolved; direct for the dose a teaspoonful. Codeia is thought to exert its influence upon the sympathetic system, without injuriously affecting the brain, circulatory or digestive apparatus. It is probable that this alkaloid, like the sugar globules, exerts its happiest effects upon the highly imaginative—its high cost, also, no doubt, contributes to its charm, for those who use it.

